# Resection of brainstem cavernous malformation via perifacial zone through retrosigmoid supracondylar approach

**DOI:** 10.3171/2019.7.FocusVid.1990

**Published:** 2019-07-01

**Authors:** Ken Matsushima, Michihiro Kohno, Helmut Bertalanffy

**Affiliations:** 1 Department of Neurosurgery, Tokyo Medical University, Tokyo, Japan; and; 2 Center for Vascular Neurosurgery, International Neuroscience Institute Hannover, Hannover, Germany

**Keywords:** cerebellopontine angle, pontomedullary sulcus, posterior fossa, transcondylar, safe entry zone, video

## Abstract

Hemorrhagic brainstem cavernous malformations carry a high risk of progressive neurological deficits owing to recurrent hemorrhages and hence require complete surgical resection while minimizing damage to the dense concentration of nuclei and fibers inside the brainstem. To access lesions inside the lower pons, the senior author (H.B.) has preferred to approach the lesions via the “perifacial zone” through the pontomedullary sulcus from the inferior surface of the pontine bulge for more than 20 years.^1,2^ This video demonstrates a case of a cavernous malformation inside the lower pons, which was surgically treated via the pontomedullary junction through the retrosigmoid supracondylar approach in a half-sitting position. The lesion was completely removed in piecemeal fashion through a tiny incision on the sulcus, which did not cause any new neurological deficits. The modified Rankin Scale improved from 4 before the surgery to 1, and the patient had no recurrence during the 2 years of follow-up. The advantage of this access and the dissection techniques for this challenging lesion are introduced, based on our experience with more than 230 surgeries of brainstem cavernoma.

The video can be found here: https://youtu.be/0H_XqkQgQ9I.

## Transcript

This video will demonstrate the microsurgical resection of a brainstem cavernous malformation via the “perifacial zone,” which is through the pontomedullary sulcus using the retrosigmoid supracondylar approach.

### 0:33 Clinical history

The patient is a 32-year-old man presenting with progressive headache, diplopia, sensory impairment on the right side, and gait disturbance. Neurological examination revealed complete left abducens paresis, hemihypesthesia of the entire right side of the face and body, and gait ataxia.

### 0:51 Preoperative radiological images

MRI displayed a 13-mm lesion inside the lower pons with a hemosiderin rim, and the so-called popcorn pattern of variable image intensities, which indicated hemorrhagic cavernoma.

### 1:06 Approach selection

The lesion was too deep to access through the middle cerebellar peduncle from the lateral surface of the pons or through the floor of the fourth ventricle from the dorsal surface of the pons. In such cases, we have preferred to approach the lesion through the pontomedullary junction from the inferior surface of the pontine bulge, which is called perifacial zone, for over 20 years. This route also provides an optimal trajectory of access along the long axis of the lesion, as we often encounter lesions on the superodorsal-to-inferoventral axis along the longitudinal fibers, including the medial lemniscus, as seen in this case.

### 1:48 Opening and exposure

The retrosigmoid supracondylar approach and access through the perifacial zone were planned with the aim of complete lesion removal under MEP, SEP, ABR, and facial electromyograms. The half-sitting position was selected to obtain optimal caudal-to-cranial viewing trajectory.

In a half-sitting position with slight head flexion, a C-shaped retroauricular incision was made. A retrosigmoid craniotomy was performed and the condylar fossa was drilled out. The dura was incised along the sigmoid sinus and caudal edge of the craniotomy.

After dural opening, the CSF was released from the cerebellomedullary cistern. The PICA, lower cranial nerves, and CNs VII and VIII were carefully dissected. The flocculus was lifted up after its dissection from the lower cranial nerves, and the choroid plexus was coagulated to decrease its size. The root exit zone of the facial nerve was precisely confirmed by electrical stimulation. Since the space between CNs IX and X did not provide adequate exposure in this case, we utilized the space between CNs X and XI. Here you can see the pontomedullary sulcus, on which the vein of the pontomedullary junction coursed. The perforating arteries were carefully separated. Clear exposure of the inferolateral surface of the pontine bulge revealed slight yellowish discoloration.

### 3:31 Lesion removal

The pia was coagulated through the space between the rootlets of CNs X and XI. About 2-mm cortical incision was made in a puncture fashion just below the exit zone of CN VII and ventral to CN IX.

The lesion cavity in the lower pons was located right underneath the incision, and the hematoma, including the fresh clot, was evacuated. The entire surface of the cavernoma was dissected at the appropriate cleavage plane from the healthy parenchyma using bipolar forceps and microscissors, and a cottonoid was placed between the separated portions of the malformation and parenchyma. As in most cases, a brain retractor was not applied to the brainstem. Piecemeal removal of the cavernoma was performed through the tiny cavity. To prevent local vasospasm, nimodipine solution was used for irrigation.

The lesion bed was carefully examined to confirm the absence of any residual cavernous malformations, which is a common cause of recurrence. You can see the size of the cortical incision and complete preservation of all the surrounding structures, including the cranial nerves, without any damage. No associated venous anomalies were seen in this case.

### 5:34 Postoperative course

Extubation was performed the next day and the patient developed no new neurological deficits. His gait disturbance disappeared within 3 weeks after the surgery.

Postoperative MRI confirmed complete resection of the lesion and significant reduction of the mass effect. Also, you can see the surgical corridor through the pontomedullary sulcus.

The patient had no new neurological deficits or recurrence during the 2 years of follow-up after the operation.

The mRS improved from 4 before the surgery to 1 on the final follow-up day.
